# Vaginal reconstruction using a gluteal transposition flap after abdominoperineal excision for anorectal malignancy

**DOI:** 10.1007/s13304-021-01211-3

**Published:** 2022-02-06

**Authors:** Björn Bolmstrand, Pehr Sommar, Per J. Nilsson, Diana Zach, Jakob Lagergren, Daniel Schain, Torbjörn Holm, Anna Martling, Gabriella Palmer

**Affiliations:** 1 Dept. of Molecular Medicine and Surgery, Karolinska Institutet and Dept. of Pelvic Surgery, Unit for Coloproctology, Karolinska University Hospital, Anna Steckséns gata 30A, D2:05, 171 76 Stockholm, Sweden; 2 Dept. of Women’s and Children’s Health, Karolinska Institutet and Dept. of Obstetrics and Gynaecology, Karolinska University Hospital, Stockholm, Sweden; 3 Dept. of Molecular Medicine and Surgery, Karolinska Institutet and Dept. of Reconstructive Plastic Surgery, Karolinska University Hospital, Stockholm, Sweden; 4 Dept. of Molecular Medicine and Surgery, Karolinska Institutet and Dept. of Surgery, Capio S:t Görans Hospital, Stockholm, Sweden; 5 Dept. of Surgery, Capio S:t Görans Hospital, Stockholm, Sweden; 6 Dept. of Molecular Medicine and Surgery, Karolinska Institutet and Dept. of Surgery, South General Hospital (Södersjukhuset), Stockholm, Sweden

**Keywords:** Rectal neoplasms, Anus neoplasms, Reconstructive surgical procedures/methods, Vagina/surgery, Surgical flaps, Postoperative complications

## Abstract

The purpose of this study is to present and evaluate a surgical method using gluteal flap for combined perineal and vaginal reconstruction after abdominoperineal excision (APE) with partial vaginectomy for anorectal malignancy. The method is a two-centre study of consecutive patients undergoing APE including partial vaginectomy for anorectal tumours, with immediate combined perineal and vaginal reconstruction using gluteal flaps. Follow-up data were retrieved via retrospective review of medical records, questionnaires and gynaecological examinations. Some 34 patients fulfilled the inclusion criteria. At the time of follow-up, 14 (78%) of the 18 patients alive responded to questionnaires. Seven (50%) of the survey responders agreed to undergo gynaecological examination. Major flap-specific complications (Clavien–Dindo > 2) were observed in 3 (9%) patients. Among survey responders, 11 (79%) had been sexually active preoperatively of which five (45%) resumed sexual activity postoperatively and three (27%) resumed vaginal intercourse. These three patients had all implemented an active vaginal health promotion strategy postoperatively. Perineo-vaginal reconstruction using gluteal flap after extended APE for anorectal malignancy is feasible. Although comparable to other methods of reconstruction, the rate of perineo-vaginal complications is high and post-operative sexual dysfunction is substantial. Postoperative strategies for vaginal health promotion may improve sexual function after vaginal reconstruction.

## Introduction

Extended abdominoperineal excision (APE) including resection of the posterior vaginal wall can be necessary to obtain clear resection margins (R0) in patients with locally advanced rectal or anal cancer. As primary closure of the vaginal defect may be insufficient for anatomical restoration, flap reconstruction is used for selected patients with the aim to restore anatomy and sexual function.

Previously reported methods for perineo-vaginal reconstruction after APE are versions of the rectus abdominis myocutaneous (RAM) flap [1–3], the gracilis flap [4–6], and, to a lesser extent, different versions of gluteal flaps [7–9].

The most probable site for vaginal involvement in locally advanced anorectal cancer is the dorsal vaginal wall. Cordeiro et al*.* have presented a classification system and a reconstructive algorithm for acquired vaginal defects [10]. Dorsal defects are classified as type 1b and the recommended flap for reconstruction is the rectus abdominis flap. Specifically, the vertical rectus abdominis myocutaneous (VRAM) flap is a frequently used technique [11]. The VRAM flap has the benefit of supplying bulky, well-vascularised and unirradiated tissue into the pelvic defect. Potential draw backs are added donor site morbidity with the risk of incisional hernia after harvest of one full rectus muscle. In addition, the transpelvic technique does not allow for minimal invasive surgery, presently performed in some institutions for highly selected patients requiring pelvic exenteration and potentially at a higher rate in the future [12, 13].

At Karolinska University Hospital where the extended or extralevator APE was pioneered, the gluteal myocutaneous flap has been the preferred method for perineal reconstruction after APE [14]. The technique previously reported has been further developed for simultaneous reconstruction of the posterior vaginal wall using an additional fasciocutaneous transposition flap.

The aim of this study is to present and evaluate this new surgical technique using gluteal flaps for reconstruction of combined perineal and posterior vaginal wall defects following extended APE for anorectal tumours.

## Methods

A retrospective cohort study on consecutive female patients undergoing APE with immediate synchronous perineal and vaginal reconstruction for anorectal malignancy at Karolinska University Hospital and Ersta Hospital, Stockholm, between January 1st 2005 and December 31st 2017 was undertaken. To minimize the risk of non-inclusion of eligible patients, two registries were used for identification: (i) an in-hospital prospective database of all operative procedures and (ii) an internal patient registry. Only patients in whom both the perineal and vaginal reconstruction was performed with a gluteal flap were included.

### Surgical technique (Fig. 1)

**Fig. 1:**
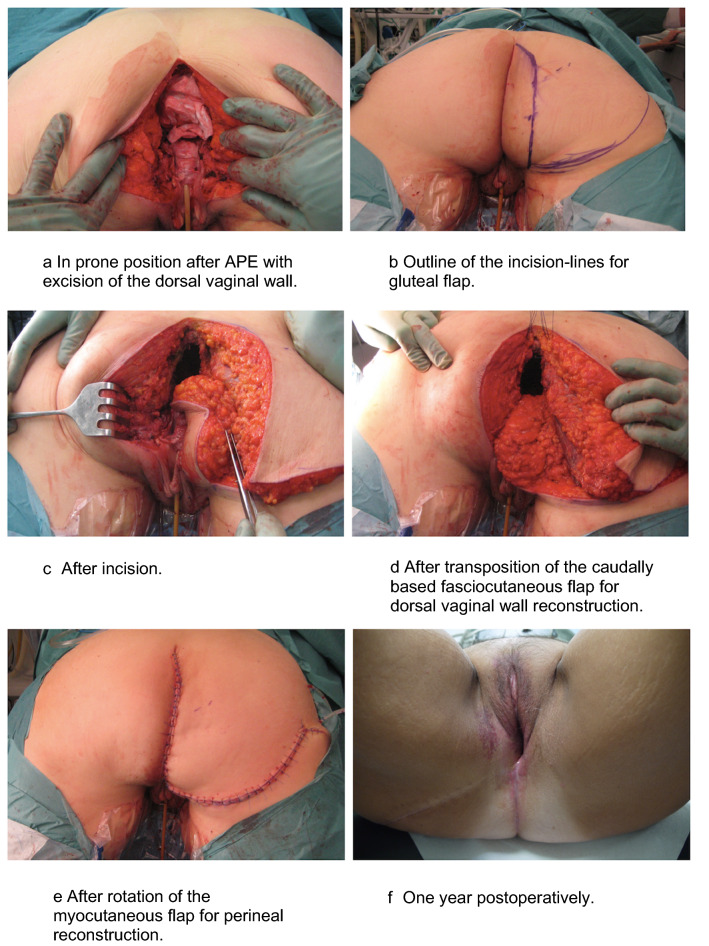
Surgical technique for immediate synchronous perineo-vaginal reconstruction using gluteal flap after APE^a^ for anorectal malignancy

Surgical resection, including *en bloc* vaginal resection, and harvest of the gluteal myocutaneous flap for perineal reconstruction is performed as previously described [14]. To make vaginal reconstruction possible, an additional caudally based fasciocutaneous transposition flap is harvested adjacent to the pelvic midline defect. The flap is designed individually for each patient according to the size of the defect, e.g., 4 cm wide at the base and 12 cm in length. Sufficient mobilization achieves reach to the posterior vaginal wall. Perforator vessels are not visualized and the base of the flap is left as a bulk of fatty tissue securing sufficient blood supply from random circulation of the inferior gluteal and pudendal vessels. After mobilization, the fasciocutaneous transposition flap is rotated 180 degrees along a cranio-caudal axis into the midline defect which allows for the gluteal skin to be used for reconstruction of the dorsal vaginal wall. When necessary, the flap is trimmed cranially to fit the defect. Using a single layer of interrupted resorbable sutures, the flap is sutured to the cut edges of the vagina. For patients in whom the most distal part of vagina is not resected and the introitus remains intact, the caudal part of the transposition flap can be deepithelialized to allow the gluteal skin in the cranial part of the flap to reach the defect in the posterior vaginal wall.

Subsequently, the cranially based myocutaneous gluteal flap is rotated over the midline defect and sutured to the opposite side gluteal muscle. Postoperatively, the patient is kept on a decubital mattress and mobilized according to a specific schedule [14].

All reconstructive surgery for patients included in this report was performed by board-certified plastic surgeons.

### Data collection

For patients included, all available in- and out-patient medical records were searched for data. Data collected included age, diagnosis, ASA score, neoadjuvant treatment, date and type of surgery, operative time (specified as time for resection and reconstruction), intensive care unit admissions, reoperations, interventional radiology procedures, post-operative treatment with antibiotics, length of hospital stay and vital status including date of death. Any complication detected within 30 days postoperatively was recorded and graded according to the Clavien–Dindo classification system of surgical complications [15]. In addition, complications related specifically to the perineo-vaginal reconstruction were recorded separately and sub-grouped into early and late complications. Early complications were defined as occurring within 30 days postoperatively and late complications when detected thereafter or persisting 90 days postoperatively.

Questionnaires were used to evaluate quality of life (QoL) and sexual function among patients alive at follow-up. For evaluation of QoL, the European Organization for Research and Treatment of Cancer general Quality of Life Questionnaire (EORTC QLQ-C30) was used. In absence of an anal cancer-specific survey, the EORTC QLQ-CR 29 (colorectal module) questionnaire was used to assess disease specific issues for all patients [16]. The Female Sexual Function Index (FSFI) was used to assess sexual function. The FSFI is a 19-item self-report survey that provides scores on overall levels of sexual function that has been validated in cancer survivors [17, 18]. A 5-point Likert scale generating an overall score is used to assess domains of female sexual function including desire, arousal, lubrication, orgasm, satisfaction, and pain. Overall score ranges from 2 to 36 and a higher score indicates a higher sexual function. A cut-off value of below 26.55 has previously been validated as indicating female sexual dysfunction [17]. As valid interpretation of the FSFI requires sexual activity within the last 4 weeks, additional set of questions were administered to assess preoperative versus post-operative sexual activity and reasons for any sexual inactivity (Appendix Table A8). Additionally, menopausal status at time of operation and whether the reconstructive surgery had been succeeded by any active strategy for vaginal health promotion was recorded in an interview setting, either in conjunction with a gynaecological examination or, for those who declined the examination, over the phone. Active vaginal health promotion was defined as systematic use of dilators, vaginal moisturizers and lubricants, pelvic floor exercises and local or systemic hormone replacement therapy (Appendix Table A9). Systemic hormone replacement alone was not considered active vaginal health promotion.

All questionnaire responders were invited to undergo a gynaecological examination that was conducted according to a pre-specified check-list (Appendix Table A10). During the examination, neovaginal elasticity, wall thickness, epithelial integrity and vascularity for both the residual vagina and the flap were recorded. In addition, neovaginal pH level, length and diameter were measured and any presence of stenosis or hair growth noted. The check-list for the gynaecological examination and the set of questions regarding vaginal health promotion were both based on versions developed and validated within The Female Sexual Medicine and Women´s Health Program at Memorial Sloan Kettering Cancer Center [19].

### Ethical approval

Ethical approval for the study was granted by the regional ethics committee of Stockholm (Regional Ethical Vetting Board, Stockholm, Sweden, Dr 2015/1547-31/4).

### Statistical analysis

Study data were analysed using the statistical software program STATA^®^ version 14.0 (StataCorp LP, College Station, TX, USA). Groups were compared with Fisher’s exact test and *p* values < 0.05 were considered statistically significant.

## Results

**Fig. 2:**
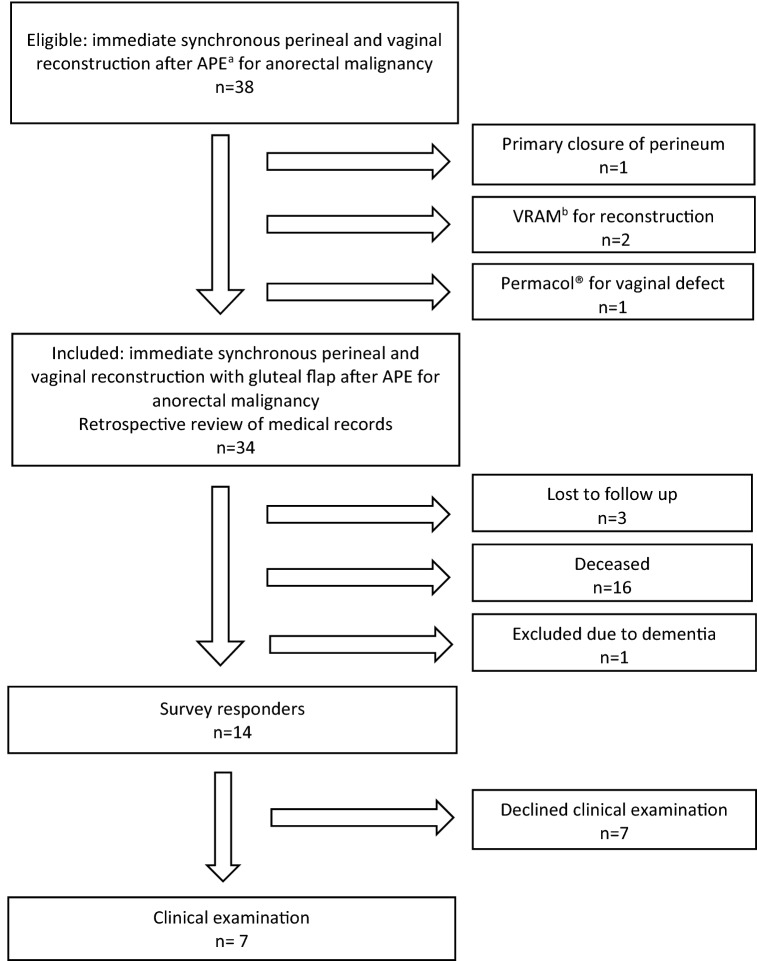
Flowchart

In total, 34 patients fulfilled the inclusion criteria during the study period. At the time of follow-up, 16 patients (47%) were deceased, one (3%) suffered from severe dementia and three (9%) did not respond to any attempts of contact. The remaining 14 patients (41%) all responded to questionnaires and seven of these patients (21%) agreed to further participation through gynaecological examination (Fig. 2.) Median follow-up time in the study was 622,5 days (8-3830+).

Patient characteristics, preoperative treatment and surgical details are presented in Table 1. All patients received pelvic external beam radiotherapy (RT) preoperatively. Among the 20 patients (59%) treated for primary rectal cancer, 13 (38%) received conventional long-course (1.8–2 Gy × 25–28) chemoradiotherapy (CRT). The remaining seven patients (21%) received short-course RT (5 Gy × 5) of whom four (12%) in combination with preoperative chemotherapy. The two patients (6%) with locally recurrent rectal cancer were initially treated with long-course RT, no re-irradiation was administered prior to surgery for the recurrent tumours. Among the 11 patients (32%) with residual or recurrent anal cancer, seven were treated with conventionally fractionated concurrent CRT in doses ranging from 46 to 60 Gy. The remaining four patients were treated with RT alone prior to salvage surgery. The single patient with a re-recurrence of anal cancer was treated with irradiation alone prior to salvage surgery and was administered chemotherapy but no re-irradiation prior to surgery for the re-recurrence.

**Table 1:** Baseline characteristics of patients undergoing synchronous primary perineo-vaginal reconstruction with a gluteal flap after APE^a^ for anorectal malignancy, *n* = 34

Variable	*
Age (years), median (range)	60 (33–83)
BMI, median (range)	23 (19–49)
ASA score	
1–2	21 (62%)
3–4	13 (38%)
Origin and presentation of cancer	
Rectal	
Primary	20 (59%)
Recurrent	2 (6%)
Anal	
Residual	8 (24%)
Recurrent	3 (9%)
Re-recurrent	1 (3%)
Neoadjuvant treatment	
Short course radiotherapy alone	3 (9%)
Short course radiotherapy w. chemotherapy	4 (12%)
Long course radiotherapy alone	5 (15%)
Chemoradiotherapy	22 (65%)

^a^Abdominoperineal excision

*Digits represent numbers of patients (%) if not specified otherwise

### Surgical details

From chart review, it is evident that 31 patients (91%) underwent extralevator APE, whereas precise surgical details for three patients (9%) were lacking. All patients underwent vaginal resection. Additional operative details are presented in Table 2. All patients underwent surgery with curative intent and histopathology reports showed that clear margins (R0) was achieved in 31 patients (91%).

**Table 2:** Surgical details in patients undergoing synchronous primary perineo-vaginal reconstruction with a gluteal flap after APE^a^ for anorectal malignancy, *n* = 34

	*
Resection beyond the TME^b^ planes	
Hysterectomy	24 (71%)
Salpingoofrectomy, unilateral	1 (3%)
Salpingoofrectomy, bilateral	22 (65%)
Sacrectomy (level S3)	1 (3%)
Extended lateral pelvic sidewall resection	1 (3%)
Lateral lymph node dissection	4 (12%)
Nephrectomy	1 (3%)
Partial resection of ureter	1 (3%)
Operative time	
Total, minutes, median (range)	541 (376–802)
Perineo-vaginal reconstruction, minutes, median (range)	139 (69–198)
R0 resection	
Rectal cancer	
Primary (*n* = 20)	20 (100%)
Recurrent (*n* = 2)	2 (100%)
Anal cancer	
Residual (*n* = 8)	7 (88%)
Recurrent (*n* = 3)	2(67%)
Re-recurrent (*n* = 1)	0 (0%)

*Digits represent numbers of patients (%) if not specified otherwise

^a^Abdominoperineal excision

^b^Total mesorectal excision

The vaginal resection included introitus in 26 patients (76%) leaving eight patients (26%) with an intact introitus. Perineal reconstruction was performed using a unilateral gluteal flap in all but one patient. For the remaining patient perineal reconstruction was performed using bilateral gluteal flaps. For vaginal reconstruction the fasciocutaneous rotational flap described above was performed in all patients. Perineo-vaginal reconstruction added a median of 139 min (69–198) to the operative time.

### Complications as revealed by review of medical records

Major complications (Clavien–Dindo > 2) including one post-operative death on day 8, occurred in nine patients (26%) (Table 3). Early complications related specifically to the perineo-vaginal reconstruction were seen in 15 patients (44%). In Table 4, details on early and late perineo-vaginal complications are presented. Only one partial flap loss was observed and necessitated a re-operation (Clavien–Dindo IIIb). The most common complication was perineal infection or dehiscence which was experienced by 13 patients (38%) with or without an underlying pelvic abscess. Two patients developed a pelvic abscess without signs of wound infection. Late perineo-vaginal complications were detected in 11 patients (32%). Among the 15 patients with early perineo-vaginal complications, eight (24%) went on to have complications at 90 days. Late complications included three patients with cutaneo-vaginal fistulas that were managed conservatively and one patient with an underlying chronic pelvic abscess who developed an entero-vaginal fistula that required re-operation.

**Table 3:** Complications according to Clavien–Dindo within 30 days postoperatively

Clavien–Dindo score	Total*	Perineo-vaginal reconstruction*
0	10 (29%)	19 (56%)
I	1 (3%)	1 (3%)
II	14 (41%)	11 (32%)
IIIa	3 (9%)	2 (6%)
IIIb	2 (6%)	1 (3%)
IVa	2 (6%)	–
IVb	1 (3%)	–
V	1(3%)	–

*Digits represent numbers of patients (%)

**Table 4:** Complications associated with the perineo-vaginal reconstruction as revealed by review of medical records (*n* = 34)

Early complications (30 days)	Number (%)	Late complications (Post 90 days)	Number (%)
Wound infection/dehiscence		Wound infection/dehiscence	
-Perineal	7 (21%)	-Perineal	2 (6%)
-Vaginal	4 (12%)	-Vaginal	1 (3%)
-Combined	2 (6%)	-Combined	1 (3%)
Partial flap loss		Fistulas	
-Perineal	1 (3%)	-Entero-vaginal	1 (3%)
-Vaginal	0 (0%)	-Cutaneo-vaginal	3 (9%)
Pelvic abscess	6 (18%)	Chronic pelvic abscess	1 (3%)
		Vaginal synechiae	1 (3%)
		Enterocele	1 (3%)
		Vaginal hair growth	1 (3%)

The perineo-vaginal complication rates for patients treated for anal and rectal cancer were 58% and 50%, respectively (*p* = 0,73).

### Gynaecological examination

Seven patients underwent the *per protocol* gynaecological examination of which five (71%) were assessed as having anatomically favourable results. In the remaining two patients (29%), partial neovaginal stenosis was observed. Median neovaginal length was 75 mm (range: 50–90 mm) and median neovaginal diameter (largest dilator not causing discomfort) was 27.5 mm (range: 20–35 mm). In all but one patient, the neovagina was assessed to have excellent to fair elasticity and no differences were observed between the residual vagina and the flap in regards to wall thickness, epithelial integrity or vascularity. Additional late complications detected at gynaecological examination were hair growth on the vaginal flap (*n* = 3) and cystocele (*n* = 1).

### Postoperative sexual function, neovaginal health promotion and QoL

Among the 14 questionnaire responders, seven had a current partner and 11 (79%) described themselves as sexually active pre-therapeutically (Table 5). Among the sexually active, five (45%) resumed some form of sexual activity post-treatment and three patients (27%) reported preserved capacity for vaginal intercourse. The 14 responders included nine rectal and five anal cancer patients. For rectal and anal cancer patients, pre-treatment sexual activity (8 vs. 3), post-treatment sexual activity (3 vs. 2), and preserved capacity for vaginal intercourse (2 vs. 1) were reported, respectively.

**Table 5:** Details on sexual function, vaginal health promotion strategies and menopausal status for survey responders, *n* = 14

Variable	Number (%)
Sexually active pre-treatment	
-Yes	11 (79%)
-No	3 (9%)
Sexually active post-treatment	
-Yes	5 (36%)
-No	9 (64%)
Vaginal intercourse post-treatment (*n* = 13)	
-Yes	3 (23%)
-No	7 (54%)
-Do not know	3 (23%)
Treatment have changed sexual enjoyment (*n* = 13)	
-Yes	9 (69%)
-No	0 (0)
-Do not know	4 (31%)
Menopausal status at time of treatment	
-Pre	3 (21%)
-Peri	2 (14%)
-Post	9 (64%)
Active implementation of vaginal health promotion strategy post-treatment	
-Yes	4 (29%)
-No	10 (71%)
Reasons stated for no implementation (*n* = 10)	
-No information	5 (50%)
-Age	1 (10%)
-No reason stated	4 (40%)
Vaginal health promotion strategy post-treatment	
-Dilator therapy	4 (29%)
-Vaginal lubricant with sexual activity	3 (21%)
-Pelvic floor exercise	4 (29%)
-Local hormonal therapy	3(21%)
-Systemic hormonal therapy	6(43%)

Among the five patients who reported resumed sexual activity post-treatment, three were sexually active at time of participation in the study. Two of these three patients scored below 26.5 points according to the FSFI, indicating sexual dysfunction. Three patients reported post-treatment sexual activity including vaginal intercourse and they had all implemented an active strategy for vaginal health promotion postoperatively. In total, less than one-third of the questionnaire responders had implemented such a strategy. Among the ten patients who had not implemented such a strategy, at least five could not recall any information in regards to vaginal health promotion.

Scale scores of the Quality of Life questionnaires are presented in Table 6 (EORTC QLQ-C30) and Table 7 (EORTC QLQ-CR29). The results indicate a substantial impairment in quality of life regarding global health status, anxiety and body image, while remaining functional scales are less affected.

**Table 6:** EORTC QLQ-C30 in patients after perineo-vaginal reconstruction with gluteal flaps after APE for anorectal malignancy, *n* = 14

Overall^a^	Mean score	(range)
Global health status	50	(0–92)
Function^a^		
Physical	70	(25–100)
Role	71	(25–100)
Emotional	72	(31–100)
Cognitive	80	(38–100)
Social	71	(25–100)
Symptoms^b^		
Fatigue	45	(0–100)
Nausea and vomiting	10	(0–100)
Pain	54	(0–100)
Dyspnoea	33	(0–100)
Insomnia	36	(0–100)
Appetite loss	12	(0–67)
Constipation	14	(0–100)
Diarrhoea	14	(0–67)
Financial difficulties	21	(0–100)

^a^Score ranges from 0 to 100; a high score represents a higher level of function

^b^Score ranges from 0 to 100; a high score represents more severe symptoms

**Table 7:** EORTC QLQ-CR29 in patients after perineo-vaginal reconstruction with gluteal flap after APE for anorectal malignancy, *n* = 14

Function^a^	Mean score	(range)
Anxiety	50	(0–100)
Body image	37	(0–100)
Sexual function (*n* = 13)	90	(67–100)
Symptoms^b^		
Micturition problems	37	(0–67)
Abdominal and pelvic pain	28	(0–78)
Defecation problems	9	(0–33)
Fecal Incontinence	36	(0–83)
Bloated feeling	31	(0–100)
Dry mouth	33	(0–100)
Hair loss	0	(0)
Trouble w. taste	7	(0–33)
Sore skin	24	(0–100)
Embarrassed by bowel movement	60	(0–100)
Stoma-related problems	17	(0–67)
Dyspareunia (*n* = 5)	40	(0–67)

^a^Score ranges from 0 to 100; a high score represents a higher level of function

^b^Score ranges from 0 to 100; a high score represents more severe symptoms

## Discussion

This report presents a novel technique for vaginal reconstruction using a gluteal fasciocutaneous transposition flap. Performed as a supplement to the gluteus maximus rotational flap, it allows for restoration of the pelvic floor and a chance to preserve sexual function after extended abdominoperineal excision with partial vaginectomy for anorectal malignancy.

In the current cohort of 34 patients, the procedure appears feasible with only one partial flap loss, an acceptable overall complication rate and reported return to sexual activity including vaginal intercourse in some patients. However, overall perineo-vaginal wound morbidity was substantial, amounting to over 50%. Perceived advantages of the described surgical method include favourable cosmesis as the incision line in the gluteal fold preserves the natural form of the buttock and the base of the transposition flap recreates a distinct transition between the perineum and the vaginal cavity. In common with the gluteus maximus rotational flap, it is easy to harvest and does not rely on pedicelled circulation.

The advancement of irradiated tissue into the perineal defect must be considered a disadvantage of this procedure. However, when comparing complication rates with those after vertical rectus abdominis myocutaneous (VRAM) flap, the difference is limited. In a study of 69 patients reconstructed with VRAM after APE for locally advanced rectal cancer, of which 47 patients were operated with combined perineo-vaginal reconstruction, the observed perineal complication rate was 36%, not including pelvic abscess formation reported in 10% of patients [20]. Other studies on VRAM in similar settings have reported perineal complication rates ranging between 46 and 50% [21, 22]. Pelvic abscess formation was more common in the current study, 18% compared to 0–10%, as reported following VRAM reconstruction [20–22]. This may, in addition to advancement of irradiated tissue, be attributed the inferior filling of the dead space within the pelvis when a gluteal flap is used compared to VRAM. It is possible that an omentoplasty, in conjunction with gluteal flap reconstruction, could mitigate these problems, but evidence is lacking [23]. Perineal herniation has been reported more frequent after gluteal flap reconstruction compared to after VRAM [24]. No perineal hernias were observed in this study.

Although sample size is limited, one can observe that perineo-vaginal complications appear to occur more often among anal cancer patients compared to those with rectal cancer. It is possible that both irradiation techniques (volume and dose) and surgical technique may increase the risk of complication for anal cancer patients [25].

The most recent systematic review indicates that restoration of sexual function after APE with partial vaginectomy for colorectal malignancy is difficult with a pooled success rate of 50% [26]. Our results point in the same direction. A separate study on sexual dysfunction after perineo-vaginal reconstruction with VRAM reports a 14% rate of return to sexual activity [27]. However, all reporting on this topic is complicated by the fact that there up until recently has been no standardized, validated method for investigating return of sexual function after extensive pelvic surgery. FSFI is validated in cancer survivors but not intended as a tool to explore reasons for sexual inactivity [17, 28, 29]. A recent study from Denmark has introduced “The Rectal Cancer Female Sexuality Score”. It has been validated among Danish women treated for rectal cancer and may prove a useful tool in the future, but was unfortunately not made available until after the current study was conceived [30].

In the literature on vaginal reconstruction, references to post-operative strategies for vaginal health promotion are almost non-existing. In a study of patients with a history of breast, gynaecological or colorectal/anal cancer, significant improvement of sexual function was observed after implementation of easy-access treatment strategies including vaginal moisturizers, vaginal lubricants, pelvic floor exercises and dilator therapy. Additional findings were improvement of vulvovaginal symptoms and less pain associated to gynaecological examinations [19]. Thus, it appears that vaginal health promotion strategies are important for cancer survivors and it may be hypothesised to be even more important following reconstruction of the vagina per se. Our finding that all three patients who resumed vaginal intercourse had implemented such a strategy is noteworthy and indicates that a formalized post-operative counselling package regarding such strategies should be implemented for all patients undergoing vaginal reconstruction. Although one cannot state that there is high-grade evidence to do so, it is highly unlikely that such a strategy would be harmful to any patient.

The current study has several limitations. Data were collected retrospectively and all relevant information may not be available in patient records and operative notes. This report is based on a series of patients where a uniform technique was applied, but no comparators are available. Despite being one of the largest cohorts to date of any one specific method for vaginal reconstruction after APE, the sample size is still small and the cohort heterogeneous regarding diagnosis, neoadjuvant treatment and time to follow-up. This makes interpretation of results difficult.

## Conclusion

Perineo-vaginal reconstruction using gluteal flap after extended APE for anorectal tumours is feasible. However, the rate of perineo-vaginal complications is high and post-operative sexual dysfunction substantial. Postoperative strategies for vaginal health promotion may improve sexual function after vaginal reconstruction.

## Figures and Tables

**Table 8:**
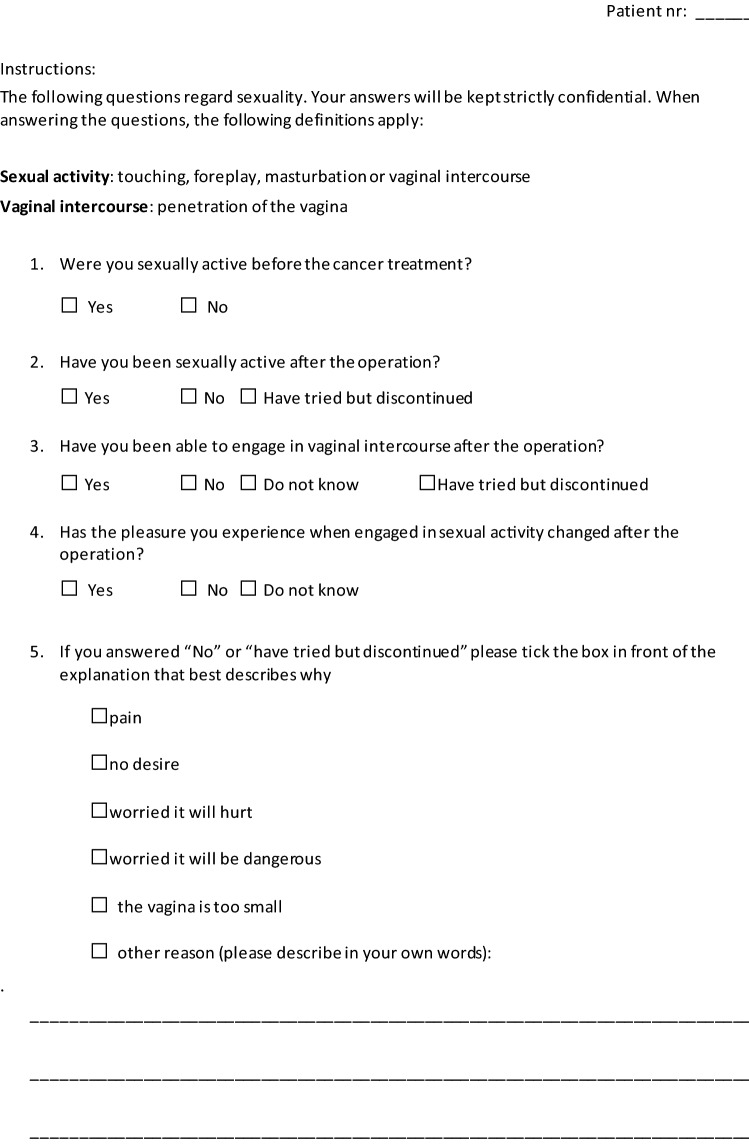
Additional set of questions to assess preoperative vs. postoperative sexual activity and reasons for any sexual inactivity

## Data Availability

The datasets generated during the study are available from the corresponding author on reasonable request.

## Appendix 1

See Appendix Tables 8, 9, 10

**Table 9:** Check-list for questions regarding vaginal health promotion strategies

Vaginal health promotion strategies^a^ (Please check appropriate response)				
Pelvic floor exercises				
Never	Rarely	A few times/w	Daily	Not applicable
Dilator therapy				
Never	Rarely	A few times/w	Daily	Not applicable
Vaginal lubricants				
Never	Rarely	A few times/w	Daily	Not applicable
Vaginal lubricants with sexual activity				
Never	Rarely	Sometimes	Always	Not active
Local estrogen treatment				
yes	no			
Systemic estrogen treatment				
yes	no			
Reason for no implementation:				
Menopausal status at time of operation				
Pre				
Peri				
Post				

^a^Modified version of check-list developed and validated within The Female Sexual Medicine and Women´s Health Program at Memorial Sloan Kettering Cancer Center

**Table 10:** Neovaginal examination check-list

Neovaginal examination check-list^a^				
pH	< 5	> 5		Not assessed
Elasticity Flap	Excellent (fully distensibility, with no or minimal tightness for speculum exam)	Fair (moderate loss of distensibility, requiring modification in speculum exam)	Poor (severe loss of distensibility, prohibiting speculum exam)	Not assessed
Elasticity Residual	Excellent (fully distensibility, with no or minimal tightness for speculum exam)	Fair (moderate loss of distensibility, requiring modification in speculum exam)	Poor (severe loss of distensibility, prohibiting speculum exam)	Not assessed
Thickness Flap	Normal (no signs of atrophy)	Thin walls	Papery thin, transparent, visible blood vessels	Not assessed
Thickness Residual	Normal (no signs of atrophy)	Thin walls	Papery thin, transparent, visible blood vessels	Not assessed
Epithelial Integrity Flap	Normal (no petechiae)	Petechiae after swabbing	Petechiae present prior to contact or bleeds w/ contact	Not assessed
Epithelial Integrity Residual	Normal (no petechiae)	Petechiae after swabbing	Petechiae present prior to contact or bleeds w/ contact	Not assessed
Vascularity Flap	Good (pink)	Fair (pale)	Minimal (no color)	Not assessed
Vascularity Residual	Good (pink)	Fair (pale)	Minimal (no color)	Not assessed
Vaginal length	mm______			Not assessed
Vaginal diameter	mm______			
Vaginal Stenosis	Complete	Partial	Not present	
Hair growth flap	Not present	Present		

^a^Modified version of check-list developed and validated within The Female Sexual Medicine and Women’s Health Program at Memorial Sloan Kettering Cancer Center
